# Cucurbitacin B inhibits HIF-1α and attenuates non-small cell lung cancer *via* ZFP91

**DOI:** 10.3389/fonc.2025.1607264

**Published:** 2025-08-11

**Authors:** Lei Song, Jing Han, Run Wang, Shen Cao, Yi Tai, Xinyu Wang, Yanjin Zheng, Shufeng Jin, Yue Xing, Hong Xiang Zuo, Ming Yue Li, Juan Ma, Xuejun Jin

**Affiliations:** ^1^ Key Laboratory of Natural Medicines of the Changbai Mountain, Ministry of Education, Molecular Medicine Research Center, College of Pharmacy, Yanbian University, Yanji, Jilin, China; ^2^ Affiliated Hospital of Yanbian University (Yanbian Hospital), Yanji, Jilin, China

**Keywords:** cucurbitacin B, non-small cell lung cancer, HIF-1α, ZFP91, anti-cancer

## Abstract

Non-small cell lung cancer (NSCLC) is among the most common types of cancer and contributes significantly to cancer-related mortality worldwide. Although some progress has been made in the treatment of NSCLC, drug resistance, high costs, and significant individual variability continue to be major obstacles to effective treatment. Cucurbitacin B (CuB) is a tetracyclic triterpenoid known for its anti-inflammatory and anticancer properties. Previous studies have demonstrated that CuB plays a therapeutic role in NSCLC, however, the mechanism by which it acts necessitates additional research. We used network pharmacology and molecular docking to predict the targets of CuB in NSCLC. Western blot, RT-PCR, immunofluorescence, and co-immunoprecipitation assays were employed to assess the therapeutic effects of CuB on NSCLC *in vitro*, as well as to elucidate the specific mechanisms involved. Cell colony formation, EDU staining, angiogenesis, scratch assays, and invasion assays, were employed to evaluate the inhibitory effect of CuB on NSCLC. *In vivo*, C57BL/6 mice were subcutaneously injected with A549 cells to establish lung cancer xenograft models for evaluating the anticancer effects of CuB. Network pharmacology revealed that the primary target of CuB for NSCLC is HIF-1α. Molecular docking studies demonstrated that CuB exhibits strong binding affinity to both HIF-1α and ZFP91. *In vitro* experiments demonstrated that CuB inhibited HIF-1α expression via ZFP91. Additionally, CuB also suppressed HIF-1α through the Akt/mTOR/p70S6K signaling pathways. Further studies demonstrated that CuB inhibited cell proliferation, migration, invasion, and angiogenesis. *In vivo* animal experiments have further demonstrated that CuB exhibits a significant therapeutic effect on lung cancer models in mice. Our experiments demonstrated for the first time that CuB inhibits HIF-1α expression through ZFP91. This inhibition reduces the proliferation, migration, invasion, and angiogenesis of lung cancer cells associated with HIF-1α. Additionally, CuB regulates HIF-1α expression by inhibiting the Akt/mTOR/p70S6K signaling pathway. Our findings further elucidate the mechanism by which CuB acts in the treatment of lung cancer, providing a rationale for its development as a therapeutic option for NSCLC.

## Introduction

Lung cancer is the leading cause of cancer-related deaths and one of the most prevalent cancers globally, accounting for approximately 1.76 million deaths and 2 million new cases each year (1). NSCLC accounts for approximately 85% of all lung cancer cases and is the predominant type of lung cancer (2). Although numerous therapeutic agents and modalities have advanced our understanding of NSCLC, challenges such as drug resistance, significant individual variability, and high costs continue to hinder its development (3). Therefore, it remains essential to explore new treatment modalities for lung cancer.

Natural compounds offer distinct advantages in disease treatment due to their structural specificity, which allows them to target multiple pathways and bind to various receptors in the human body. Structural diversity also makes natural compounds less prone to drug resistance. CuB is a natural compound extracted from Cucurbitaceae plants. It has anti-inflammatory, immunomodulatory effects, and cancer treatment potential (4). Although numerous studies have demonstrated that CuB can inhibit NSCLC through various pathways (5–8), the precise mechanism by which CuB exerts its inhibitory effects on NSCLC requires further investigation.

HIF-1α was discovered in the early 1990s by *Gregg L. Semenza* and colleagues, and he eventually received the Nobel Prize for this work. During tumorigenesis, most tumor cells, even in the presence of oxygen, preferentially generate energy through the glycolytic pathway. This process leads to the accumulation of lactate, the product of glycolysis, within the cells. As a result, local acidification occurs (indicated by a drop in pH), creating a hypoxic environment due to the substantial energy demands required to sustain their growth (9). In hypoxia, HIF-1α activation is crucial for multiple tumor development processes, including proliferation, apoptosis, invasion, angiogenesis, metabolism, immunity, and treatment resistance (10). CuB exerts an inhibitory effect on HIF-1α (11); nevertheless, the specific contribution of this inhibition to the progression of NSCLC remains to be elucidated.

Zinc finger protein 91 (ZFP91) is a 63.5 kDa nuclear protein that is conserved and possesses structural features characteristic of transcription factors. Research indicates a correlation between ZFP91 and the pathogenesis of colorectal, prostate, breast, and gastric cancers (12–15). However, it remains unclear whether ZFP91 is involved in the pathogenesis of lung cancer. Previous studies have shown ZFP91 activates HIF-1α via the NF-κB/p65 pathway, promoting colon cancer development (16). Therefore, this paper investigates the involvement of ZFP91 and HIF-1α in the pathogenesis of NSCLC.

Our study identified potential targets of CuB in NSCLC using network pharmacology and molecular docking, followed by experimental validation. We first demonstrated that CuB inhibits HIF-1α through ZFP91, reduces the proliferation, migration, and invasion of A549 cells, and inhibits tubule formation in HUVEC cells. The results suggest that CuB has potential therapeutic efficacy in NSCLC.

## Materials and methods

### Network pharmacology predicted targets

The drug targets of CuB were identified using an organic small molecule bioactive data analysis platform (PubChem) and a pharmacophore matching and potential recognition target platform (PharmMapper). Associated targets for NSCLC were sourced from the Disease Gene Association Database (DisGeNET) and the Human Gene Database (GeneCards). Venny 2.1 plots Venn diagrams of CuB versus NSCLC. Cytoscape 3.7.1 and the String database were utilized to construct a protein-protein interaction (PPI) network for CuB-NSCLC interactions, and confidence score thresholds ≥ 0.4 were set to select high-confidence core genes. The DAVID database conducts KEGG signaling pathway enrichment analysis on intersecting genes.

### Molecular docking experiment

The PubChem database retrieves SDF format files for CuB. Key target protein structures were obtained from the Protein Data Bank (PDB; http://rcsb.org/pdb).The target protein, including water molecules and small molecule ligands, was optimized using PyMOL 2.1.0. Molecular docking was performed with AutoDock Tools 1.5.6, and results were analyzed and visualized using Discovery Studio 2019 Client.

### Cell culture and reagents

American type culture collection (ATCC, Manassas, VA, USA) supplied A549, HeLa, Hep3B, HUVEC, HCT116 and HEK293T cells. A549, HeLa, Hep3B, HEK293T and HUVEC cells were grown in DMEM medium (BDBIO, Hangzhou, China). HCT116 cells were cultured in RPMI-1640 medium at 37°C with 5% CO2. A549 cells were activated with 10 ng/mL TNF-α for 12 h to induce HIF-1α.

TNF-α was sourced from R&D Systems, USA. CuB (>98% purity) was obtained from Chengdu Herbpurify Co., Ltd, China, prepared as a 30 mM DMSO solution, and stored at -20°C.

### Plasmids and siRNA transfection

A549 and HEK293T cells were transfected with siRNAs and overexpression plasmids using Lipofectamine 2000 for 24 h. The plasmids (Flag-ZFP91, Flag-HIF-1α) and siRNAs (si-HIF-1α from GeneChem, si-ZFP91 from Bioneer) were utilized as indicated.

### Cell viability assay

Cells were seeded in 96-well plates at a density of 5×10^3^ cells per well. And cultured for 12 h. Afterward, the cells were co-cultured with DMSO and various concentrations of CuB for 24 h. Following incubation, 10 μL of MTT was added per well and incubated at 37°C with 5% CO2 for 4 h. DMSO solubilized the formazan crystals, and absorbance was assayed at 570 nm with Multiskan GO.

### Western blotting analysis

Protein cleavage and extraction methods were conducted as previously described (17). The primary antibodies used in this study included: Santa Cruz: cyclin D1 (sc-8396, 1:1000), c-Myc (sc-789, 1:1000), Topo-1 (sc-137238, 1:1000), GAPDH (sc-365062, 1:1000), AKT (sc-1618, 1:1000), mTOR (sc-517464, 1:1000), p70S6K (sc-8418, 1:1000), 4EBP1 (sc-293124, 1:1000), eIF4E (sc-9976, 1:1000), VEGF (sc-507, 1:1000), and MMP-9 (sc-6840, 1:1000). CST Company: HIF-1β (5537, 1:1000), p-AKT (C060, 1:1000), p-mTOR (2971, 1:1000), p-p70S6K (9234, 1:1000), p-4EBP1 (2855, 1:1000), and p-eIF4E (9741, 1:1000). Abmart Corporation: HA-Tag (M20003, 1:4000). Sigma: Flag-Tag (F-2922, 1:500). BETHYL Corporation: ZFP91 (A303-245A, 1:1000).

### RT-PCR

Trizol solution was used to extract total RNA, and the Roche Transcriptor First Strand cDNA Synthesis Kit was utilized for cDNA synthesis. Subsequently, the TaKaRa Taq™ RT-PCR Kit was utilized for sample amplification. The PCR products were analyzed on a 3% agarose gel stained with 5 μL of DNAGREEN, and the bands were visualized under UV light. The primers utilized are detailed in **Table 1**.

**Table 1:** RT-PCR forward and reverse primers.

Primer	Sequence
HIF-1α	5′-CAAGTCAGCAACGTGGAAGG-3′(forward)
	5′-ATCAGCACCAAGCACGTCAT-3′(reverse)
ZFP91	5′-TTAATGGGGTACAGGGGTCTC-3′(forward)
	5′-TCTATTCCAGAAACCTGGCA-3′(reverse)
Cyclin-D1	5′-GTAGCAGCGAGCAGCAGCAGAGT-3′ (forward)
	5′-CTCCTCGCACTTCTGTTCCTC-3′ (reverse)
VEGF	5′-GCTCTACCTCCACCATGCCAA-3′ (forward)
	5′-TGGAAGATGT-CCACCAGGGTC-3′ (reverse)
MMP-9	5‘-CAACATCACCTATTGGATCC-3’ (forward)
	5‘-CGGGTGTAGAGTCTCTCGCT-3’ (reverse)
GAPDH	5′-ACCACAGTCCATGCCATCAC-3′ (forward)
	5′-TCCACCACCCTGTTGCTGTA-3′ (reverse)
c-Myc	5′-GGTCTATATAAGCAGAGCTA-3′ (forward)
	5′-GTGGTATGGCTGATTATGATCAG-3′ (reverse)

### Immunoprecipitation

Total cellular protein was extracted using immunoprecipitation buffer, and supernatants were collected and incubated with 20 μL of agarose beads (sc-2003, Santa Cruz Biotechnology) and 2 μL of specific antibodies at 4°C for 12 h. Following centrifugation, the agarose beads were washed with immunoprecipitation buffer. The immunoprecipitated complexes were then analyzed by Western blotting.

### Immunofluorescence

A549 cells were seeded in 24-well plates, fixed with 4% paraformaldehyde, permeabilized with 0.2% Triton X-100, and blocked with 5% BSA. The cells were then incubated overnight with primary antibodies, treated with fluorescent secondary antibodies for 30 min, stained with DAPI to visualize nuclei, and observed using confocal microscopy.

### Colony formation assay

A549 cells were cultured in 6-well plates at 2×10² cells/well for 14 days, then fixed with 10% paraformaldehyde. Colony formation was stained with 1% crystal violet and photographed for analysis.

### EdU assay

Proliferating A549 cells were labeled with the EdU Kit (RIBOBIO, Guangzhou, China) after 6 h of co-culture in 96-well plates. Subsequently, the operation was carried out in accordance with the reagent instructions.

### Scratch assay

A549 cells were seeded in 24-well plates and a straight-line scratch was created on the monolayer after 12 h using a sterile pipette tip. The cells were then washed with PBS to remove debris and cultured as usual. After 24 h, the migration rate of the cells was observed and the width of the scratch was compared to the final and initial widths.

### Matrigel transwell invasion assay

Matrigel was diluted 1:8 with serum-free medium at 4°C and polymerized in the upper chamber of the Transwell after 3 h at 37°C. A549 cells were suspended in serum-free medium and loaded into the upper compartment of a transwell apparatus, while the lower chamber contained complete growth medium. Following 24-hour incubation under standard conditions (37°C, 5% CO_2_), stationary cells on the membrane’s apical surface were carefully eliminated. Migrated cells adherent to the basolateral side underwent sequential processing: 4% paraformaldehyde fixation, 0.1% crystal violet nuclear staining, and microscopic enumeration at 200× magnification.

### Tube formation assays

First, spread 300 μl Matrigel evenly on the upper chamber of Transwell at 4°C, put it into 24-well plate and incubate it in the incubator at 37°C for 1 h. HUVEC cells were resuspended in complete medium, seeded in 24-well plates, and incubated. Once tubular structures were evident, cells were imaged and analyzed under a fluorescence microscope.

### Mice

Athymic BALB/c male nude mice (20 ± 2 g, 4–5 weeks old) were obtained from Life River Laboratory Animal Co., Ltd., Beijing, China. The mice were randomly divided into four groups (n = 5 per group): a control group, a 0.1 mg/kg CuB group, a 0.25 mg/kg CuB group, and a 0.5 mg/kg CuB group. A549 cells (6.5 × 10^7^) were subcutaneously implanted into the left flank of nude mice. Tumors developed 7 days post-injection. Mice were orally administered CuB in normal saline every 3 days for 30 days. Solid tumors were harvested from euthanized animals and used for Western blotting.

### Statistical analysis

Statistical analyses were presented as mean ± SD (n ≥ 3) and analyzed using one-way ANOVA with Tukey’s multiple comparison tests (Graphpad Software). Significance was set at *p < 0.05*.

## Results

### Network pharmacology predicts critical targets for CuB in NSCLC

**Figure 1:**
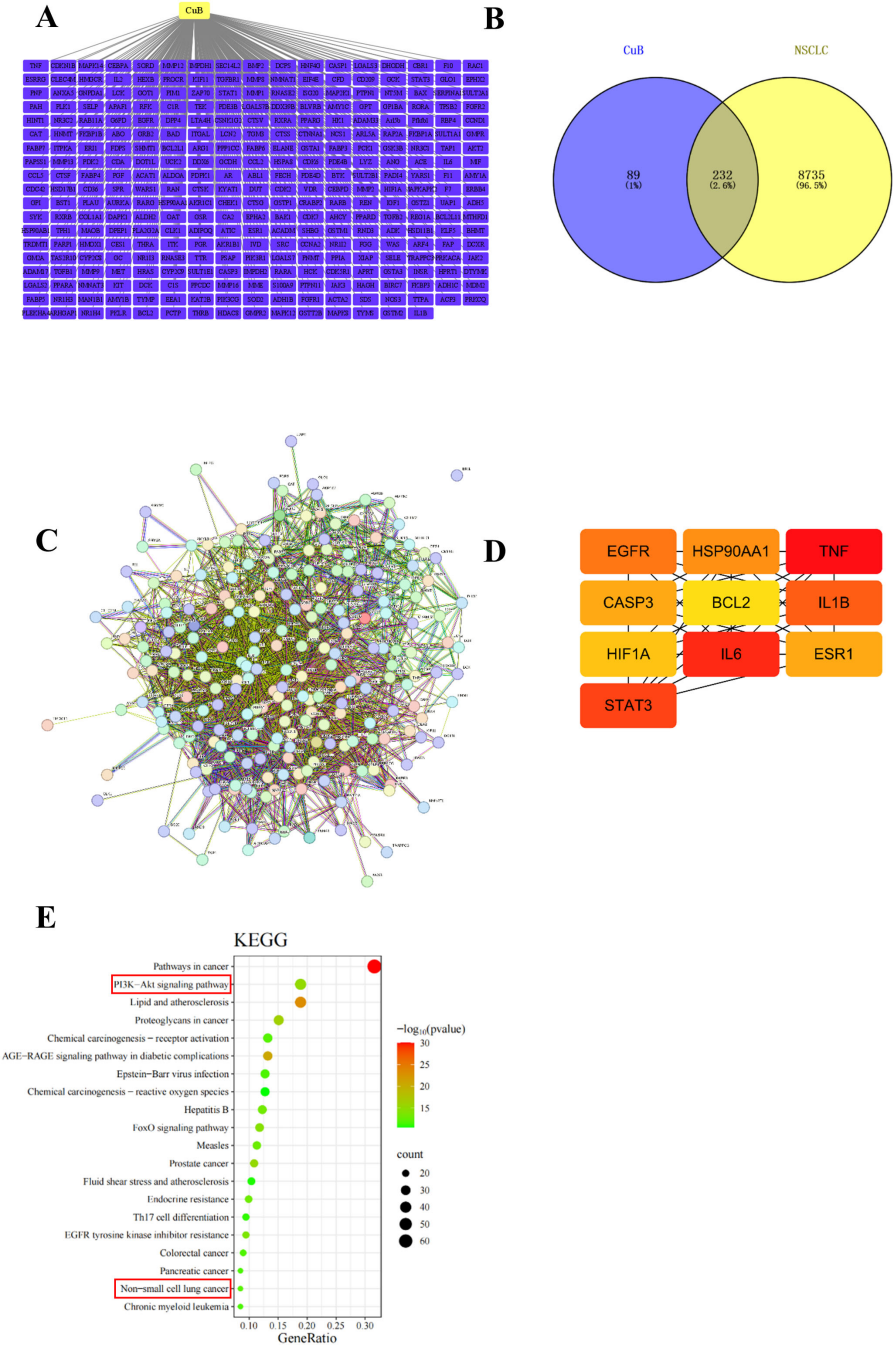
Network pharmacology for predicting core targets of CuB in NSCLC. **(A)** gene network with CuB linking to multiple genes; **(B)** Venn diagram showing overlap between CuB and NSCLC genes; **(C)** complex gene interaction network; **(D)** simplified gene interaction model highlighting key genes such as HIF-1α; **(E)** KEGG pathway analysis bar graph illustrating significant pathways, including cancer and signaling pathways, with varying levels of significance represented by color gradient.

To investigate whether CuB has a therapeutic effect on NSCLC, we initially employed network pharmacology to predict potential targets of CuB that may be effective in treating NSCLC. The pharmacodynamic targets of CuB are illustrated in **Figure 1A**. The results of Venny plot showed that there were 232 intersection targets of CuB with NSCLC **Figure 1B**. The PPI network was constructed using the STRING database and Cytoscape version 3.7.1 software (**Figure 1C**). A confidence score threshold of ≥ 0.4 was established to select core genes with high confidence (**Figure 1D**). The results showed that HIF-1α is a key target of CuB in the treatment of NSCLC. KEGG signaling pathway enrichment was subsequently conducted using the DAVID database (**Figure 1E**). The results indicate that signaling pathways, including those related to NSCLC and the PI3K/AKT pathway, are associated with the intersection genes.

**Figure 2:**
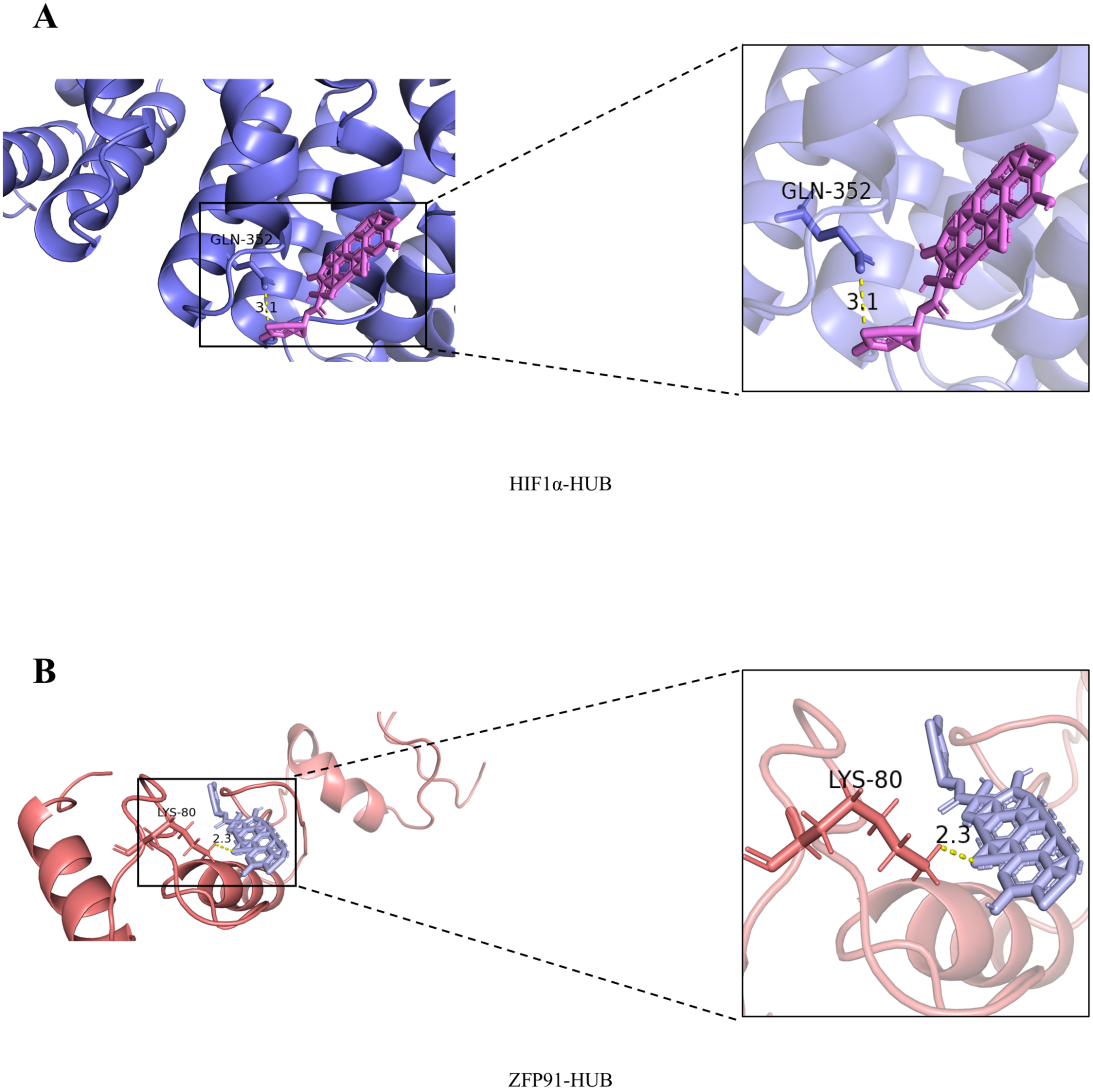
Molecular docking results of CuB with HIF-1α and ZFP91. **(A)** Molecular Docking Results of CuB with HIF-1α. **(B)** Molecular Docking Results of CuB with ZFP91.

HIF-1α plays a critical role in various types of cancer, including NSCLC (18). Therefore, we tested whether CuB could bind to HIF-1α using molecular docking techniques. Our results demonstrated that CuB exhibited strong binding affinity to the binding pocket of HIF-1α, forming an interaction with the GLN-352 residue of HIF-1α (**Figure 2A**). Previous studies have demonstrated that the overexpression of ZFP91 results in enhanced activation of the NF-κB signaling pathway, which is dependent on the presence of NF-κB-inducing kinase (NIK). Furthermore, NIK activity is linked to the pathogenesis of lung cancer (19); therefore, ZFP91 may contribute to the development of this disease. To investigate whether the effect of CuB on NSCLC is associated with ZFP91, we examined the binding interaction between CuB and ZFP91. Our findings indicate that CuB can bind to the LYS-80 residue of ZFP91 (**Figure 2B**). These results suggest that the function of CuB may be associated with HIF-1α and ZFP91.

### CuB suppressed the protein expression of ZFP91 and HIF-1α

**Figure 3:**
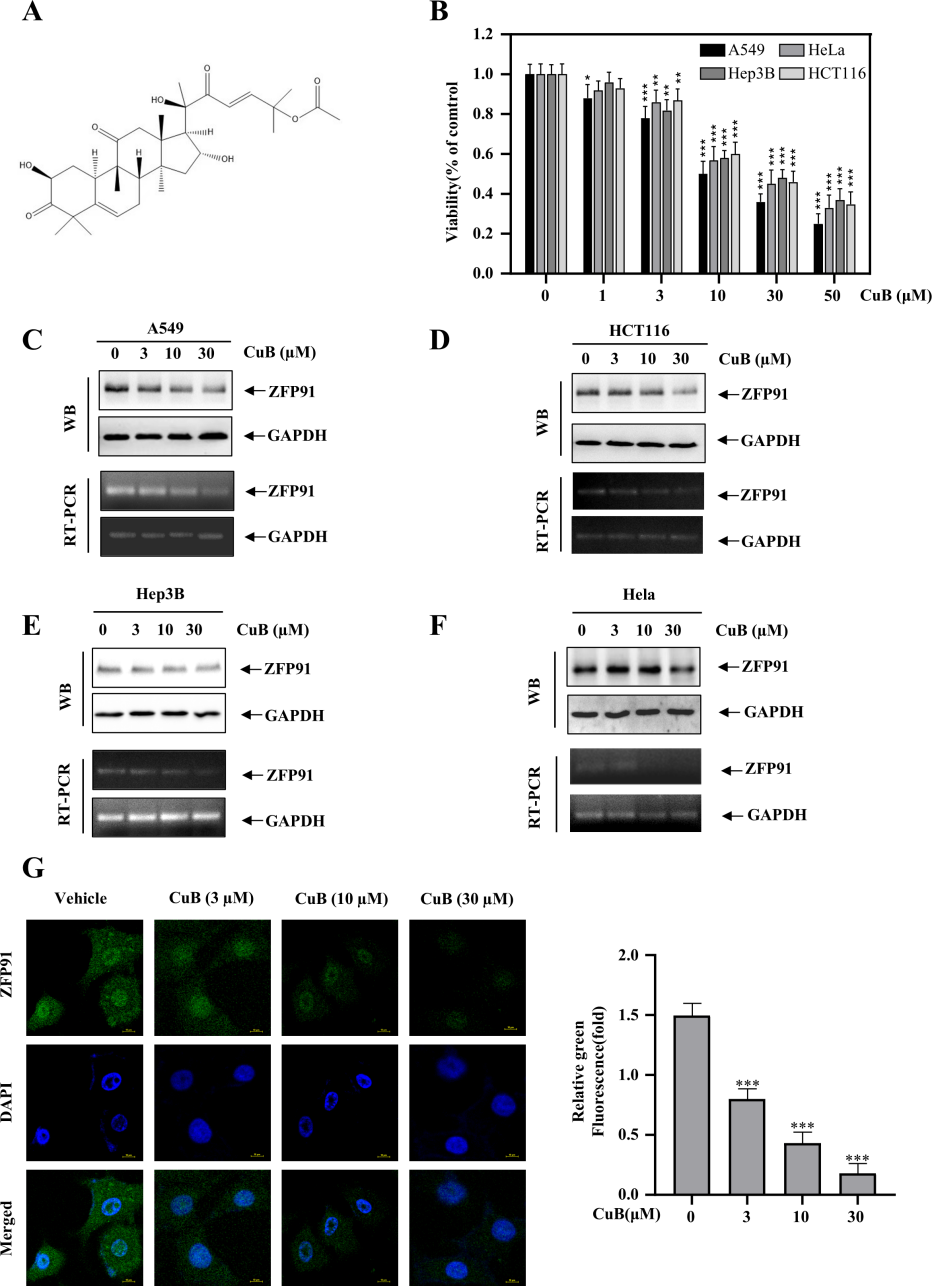
CuB inhibits the protein and mRNA expression of ZFP91. **(A)** Chemical Formula of CuB. **(B)** The MTT assay was used to evaluate CuB’s effect on A549 cell viability. **(C-F)** Effect of CuB on protein and mRNA expression of ZFP91 in A549, HCT116, Hep3B, and HeLa Cells. **(G)** Effect of CuB on ZFP91 fluorescence intensity in A549 cells. Original magnification: 600×. Data are represented as mean ± standard, * p<0.05, ** p<0.01, *** p<0.001 compared with Control group.

**Figure 3A** presents the chemical structure of CuB. CuB has been reported to exhibit a significant therapeutic effect against various cancers. We validated its efficacy using the MTT assay across four cell lines: A549, HeLa, Hep3B, and HCT116. The results indicated that CuB significantly inhibited cell viability in all tested cell lines. Among the compounds tested, CuB exhibited the most significant inhibitory effect on the A549 cell line (**Figure 3B**). ZFP91 has been reported to play a significant role in various cancers. Our Western blot and RT-PCR analyses demonstrated that ZFP91 was highly expressed in A549, HCT116, Hep3B, and HeLa cells. In addition, CuB was found to inhibit both protein and mRNA expression of ZFP91 in four cell lines, with the most pronounced effect observed in A549 cells (**Figure 3C-F**). These results indicate that CuB can exert an inhibitory effect on A549 cells, potentially mediated by ZFP91. In addition, immunofluorescence experiments demonstrated that CuB suppressed ZFP91 expression in A549 cells (**Figure 3G**).

**Figure 4:**
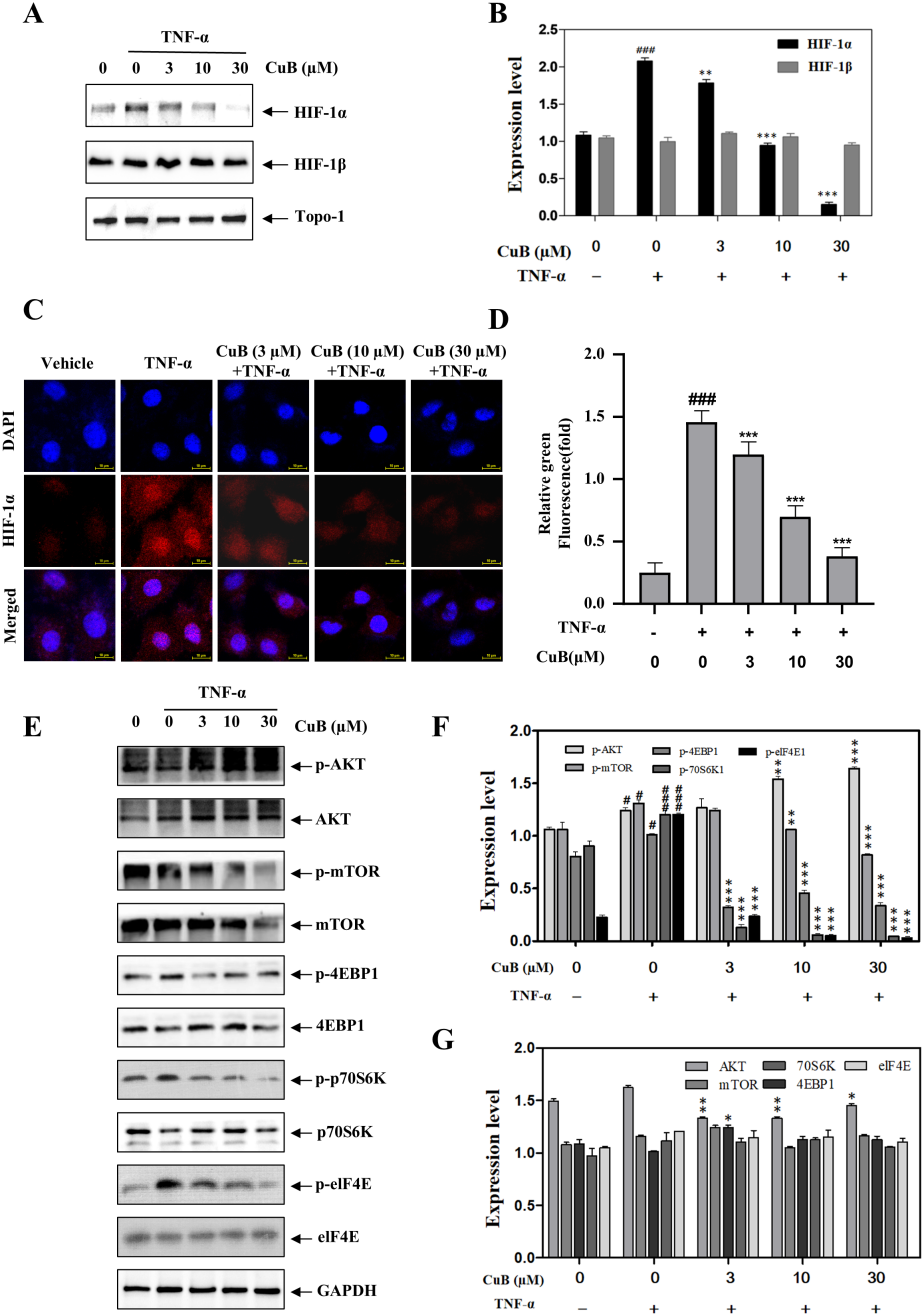
CuB inhibits the protein and mRNA expression of HIF-1α. **(A, B)** The effect of CuB on HIF-1α protein expression **(A)** and the corresponding statistical analysis **(B)**. Protein expression of HIF-1α was evaluated in A549 cells. **(C, D)** Effect of CuB on HIF-1α fluorescence intensity in A549 cells. Magnification: 600×. **(E-G)** Effect of CuB on the epression of HIF-1α-related pathway proteins and its statistical analysis. Data are mean ± SD, *p< 0.05, **p< 0.01, ***p<0.001 vs. TNF-α group.

HIF-1α is pivotal in tumor progression, previous studies have shown that the expression of HIF-1α in lung cancer tissues is significantly higher than that in normal lung tissues, and its high expression promotes a series of reactions such as tumor proliferation, migration, and invasion, and this process is associated with the PI3K/AKT/mTOR signaling pathway (20, 21). Therefore, we investigated whether CuB could affect proteins such as HIF-1α, the PI3K/AKT/mTOR pathway, and their downstream targets, p70S6K and eIF4E, in A549 cells. Our investigation in A549 cells revealed CuB-mediated suppression of HIF-1α protein expression (**Figures 4A, B**). Furthermore, immunofluorescence experiments provided additional validation for our conclusions (**Figures 4C, D**). In addition, CuB also inhibited the upstream signaling events of the PI3K/AKT/mTOR pathway related to HIF-1α, as well as the protein expression of p70S6K, eIF4E, and 4EBP1 (**Figures 4E–G**).

### CuB inhibits the expression of HIF-1α through ZFP91

**Figure 5:**
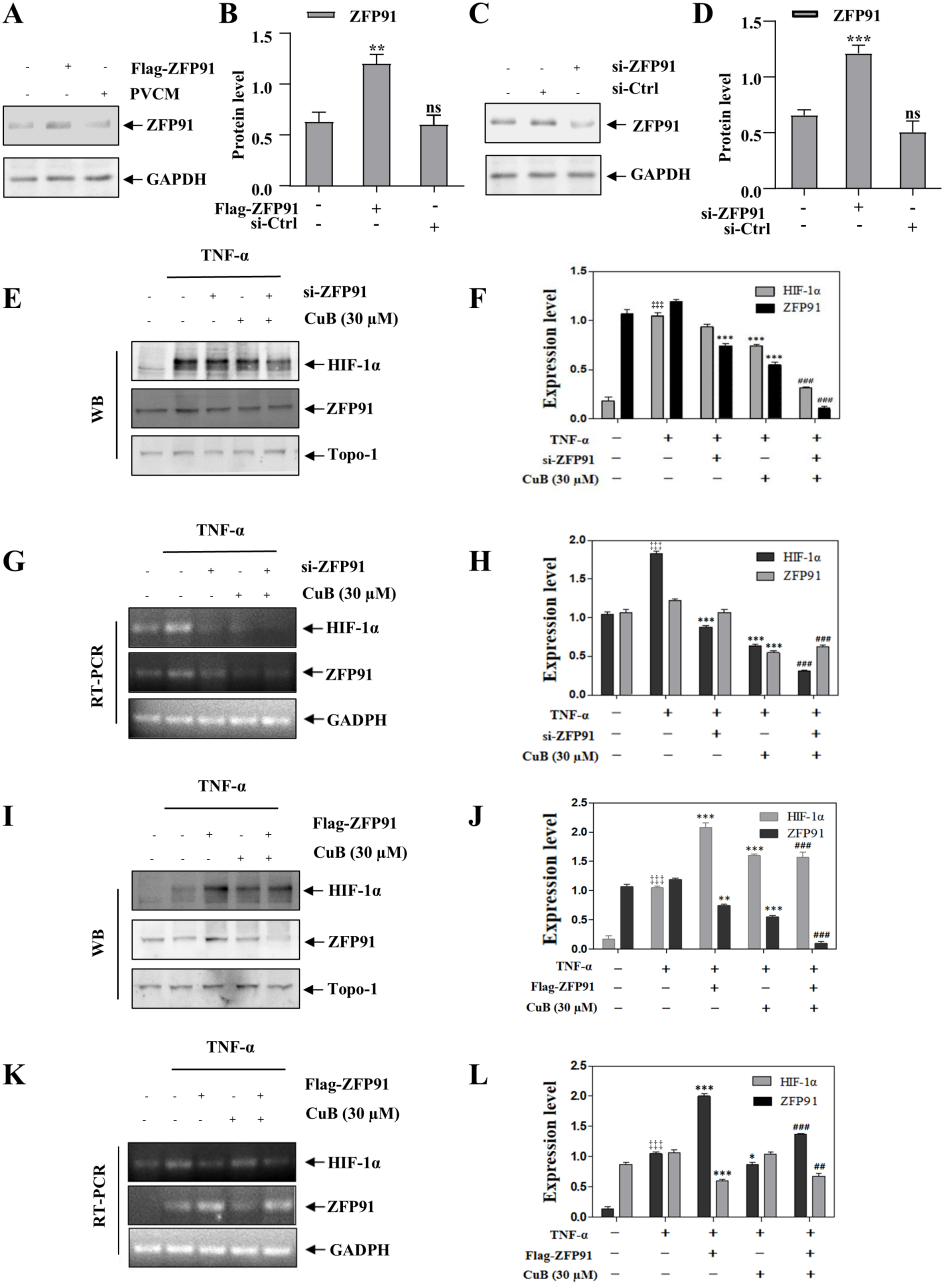
CuB inhibits the protein expression of HIF-1α through ZFP91. **(A-D)** Two plasmids, Flag-ZFP91 and si-ZFP91, were transfected into A549 cells for 48 h. Western blot analysis was conducted to evaluate the effects of overexpressing or silencing ZFP91. Data are mean ± SD, ***p<0.001 vs. Control group. **(E–L)** After the overexpression or silencing of ZFP91, the protein and mRNA expression levels of HIF-1α were assessed using Western blotting and RT-PCR. Data are mean ± SD, ^‡‡‡^ *p<0.001* vs. Control group, *p< 0.05, **p< 0.01, ***p<0.001 vs. TNF-α group. *^##^p< 0.01, ^###^p<0.001* vs. TNF-α + CuB group.

**Figure 6:**
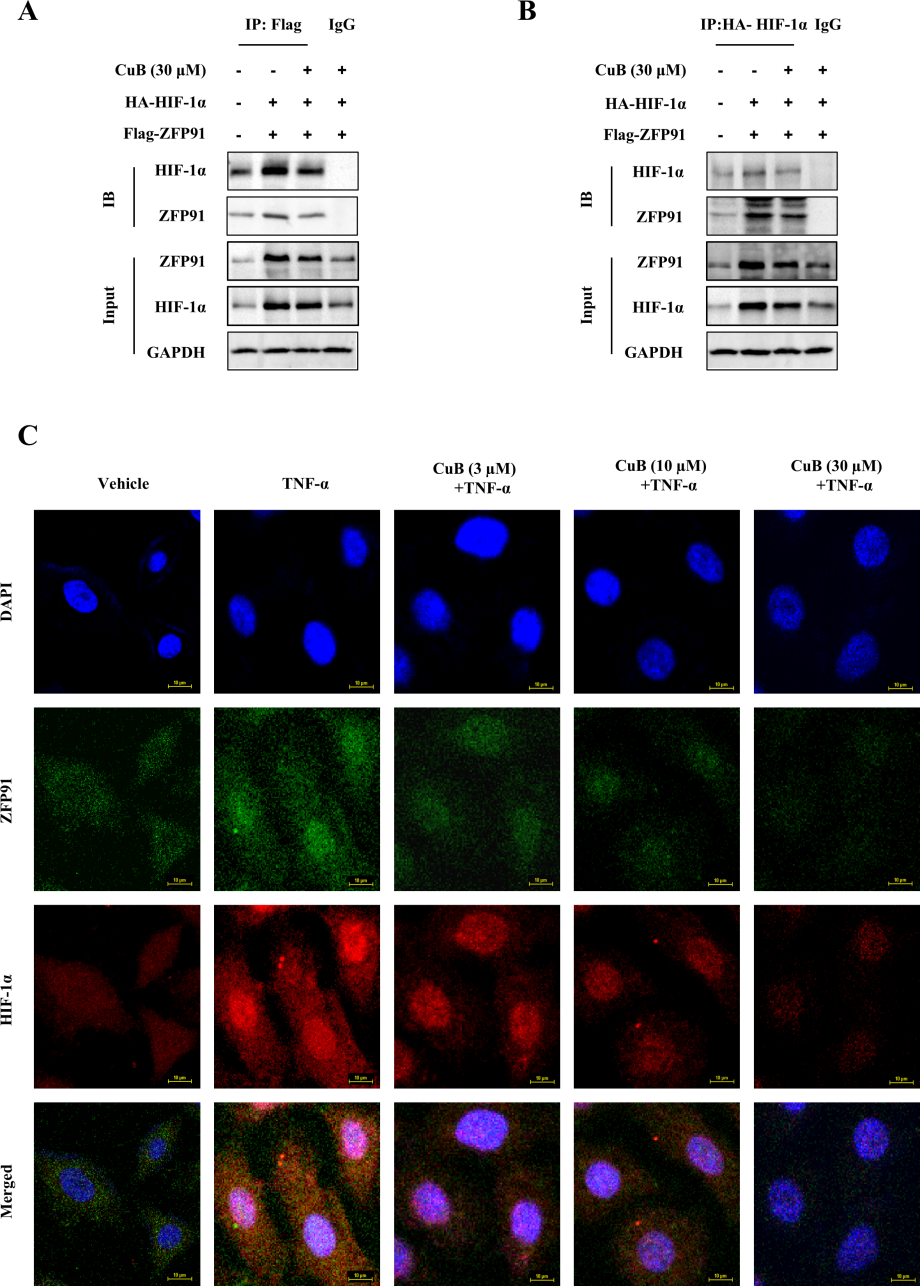
CuB inhibited the interaction between ZFP91 and HIF-1α. **(A, B)** A549 cells were transfected with IP: HA-HIF-1α and Flag-ZNF91 plasmids for. Co-immunoprecipitation was then performed to pull down either Flag-ZNF91 or IP: HA-HIF-1α. **(C)** Co-localization of ZFP91 with HIF-1α within the cells was detected using immunofluorescence. Magnification: 600×.

Our study demonstrated that CuB suppressed ZFP91 and HIF-1α expression in A549 cells, though their functional interplay in lung cancer remains to be elucidated. Therefore, in our subsequent experiments, we overexpressed and silenced ZFP91 in A549 cells, respectively (**Figures 5A-D**). Our results demonstrated that the overexpression of ZFP91 significantly upregulated both the protein and mRNA expression levels of HIF-1α. Conversely, the silencing of ZFP91 resulted in a decrease in the protein and mRNA expression levels of HIF-1α. On this basis, the expression of HIF-1α protein and mRNA was further inhibited by CuB (**Figures 5E-L**). In addition, co-immunoprecipitation experiments demonstrated an interaction between ZFP91 and HIF-1α; however, the addition of CuB was found to attenuate this interaction (**Figures 6A, B**). Further, immunofluorescence analysis demonstrated that CuB reduced the co-localization of ZFP91 with HIF-1α in cells (**Figure 6C**). These results indicate that ZFP91 exerts its function through interaction with HIF-1α, while CuB inhibits this interaction and also suppresses their co-localization in cells.

### CuB inhibited the protein expression of c-Myc, Cyclin D1, VEGF, and MMP-9 via ZFP91

Angiogenesis and dysregulation of the cell cycle are critical steps in tumor development, with HIF-1α playing a significant role in this process (22, 23). Our previous results preliminarily demonstrated that the inhibitory effect of CuB on HIF-1α is correlated with ZFP91. Therefore, to further investigate the regulation of HIF-1α by CuB through ZFP91, we overexpressed and silenced ZFP91 in the subsequent experiment to observe the impact of these manipulations on cell cycle progression and angiogenesis-related proteins.

**Figure 7:**
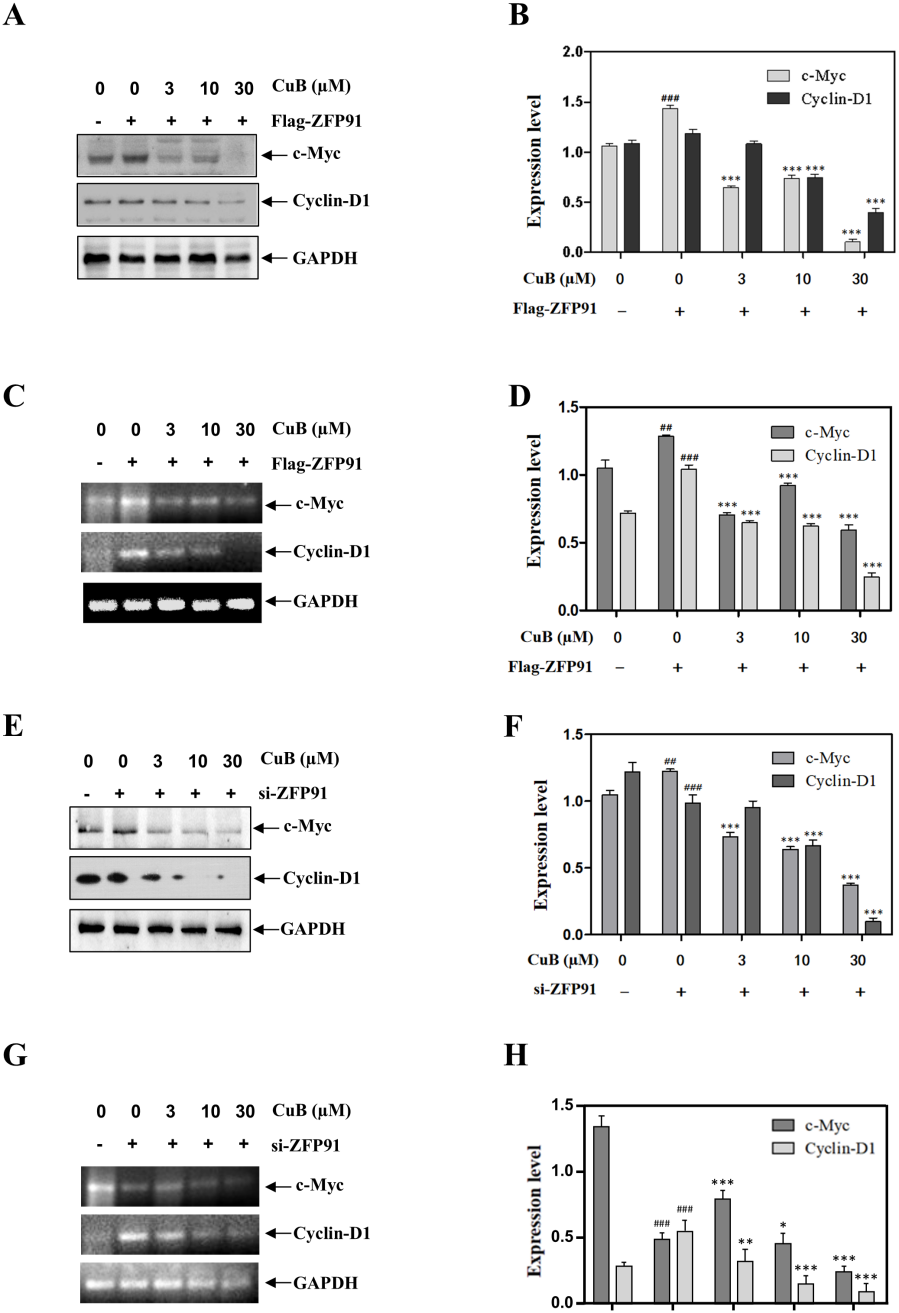
CuB inhibited the expression of c-Myc and Cyclin D1 proteins by ZFP91. **(A-H)** Protein and mRNA levels of c-Myc and Cyclin D1 were assessed by Western blotting and RT-PCR. Data are mean ± SD, *^##^p*< 0.01*, ^###^p*<0.001 vs. Control group, *p< 0.05, **p< 0.01, ***p<0.001 vs. Flag-ZFP91 group or si-ZFP91.

Our results demonstrated that the overexpression of ZFP91 significantly increased both the protein and mRNA levels of c-Myc and Cyclin D1. This effect was reversed with the addition of CuB (**Figures 7A-D**). When we silenced ZFP91, both the protein and mRNA expression levels of c-Myc and Cyclin D1 decreased. Additionally, the introduction of CuB further reduced their expression (**Figures 7E-H**).

**Figure 8:**
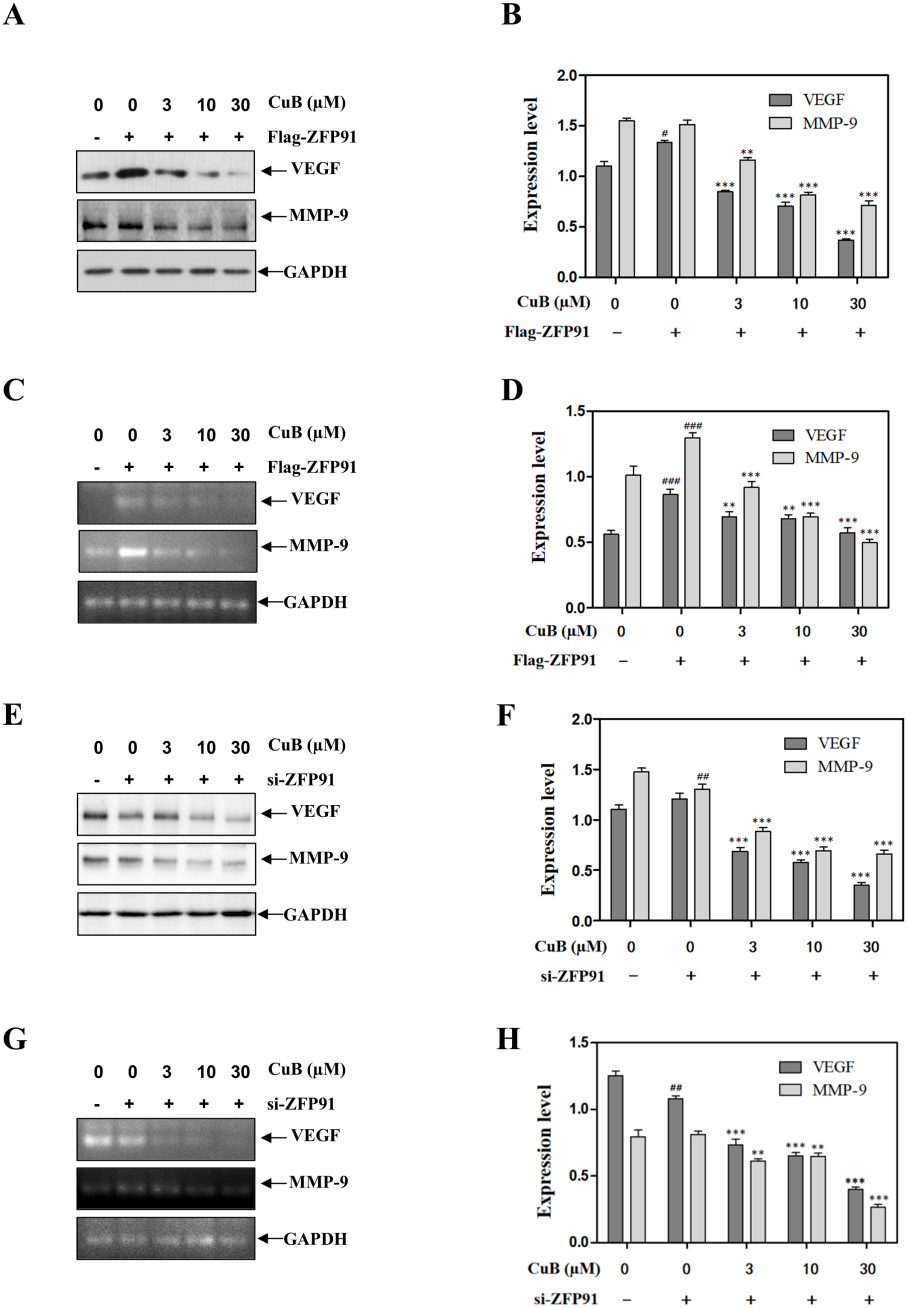
CuB inhibited the expression of VEGF and mmp-9 proteins by ZFP91. **(A-H)** Protein and mRNA levels of VEGF and MMP-9 were assessed by Western blotting and RT-PCR. Data are mean ± SD, *^#^p< *0.05*, ^##^p<* 0.01, *^###^p<*0.001 vs. Control group, *p< 0.05, **p< 0.01, ***p<0.001 vs. Flag-ZFP91 group or si-ZFP91.

Similar to the results mentioned above, the overexpression of ZFP91 led to an increase in both protein and mRNA levels of VEGF and MMP-9. Conversely, ZFP91 silencing downregulated VEGF and MMP-9 expression. Additionally, the introduction of CuB also reduced the expression of VEGF and MMP-9 (**Figures 8A-H**). These results suggest that CuB may exert anticancer activity by modulating genes involved in lung cancer downstream of HIF-α through ZFP91.

### CuB functions by inhibiting proliferation, migration, invasion, and angiogenesis

Our previous studies have demonstrated that CuB can regulate HIF-1α via ZFP91. To further investigate this effect, we overexpressed or silenced HIF-1α and ZFP91 in subsequent experiments and administered CuB to assess its inhibitory effects on proliferation, migration, invasion, and tubule formation. Cell colony assays demonstrated that the knockdown of ZFP91 or HIF-1α led to a decrease in colony numbers, which were further diminished following treatment with CuB.

**Figure 9:**
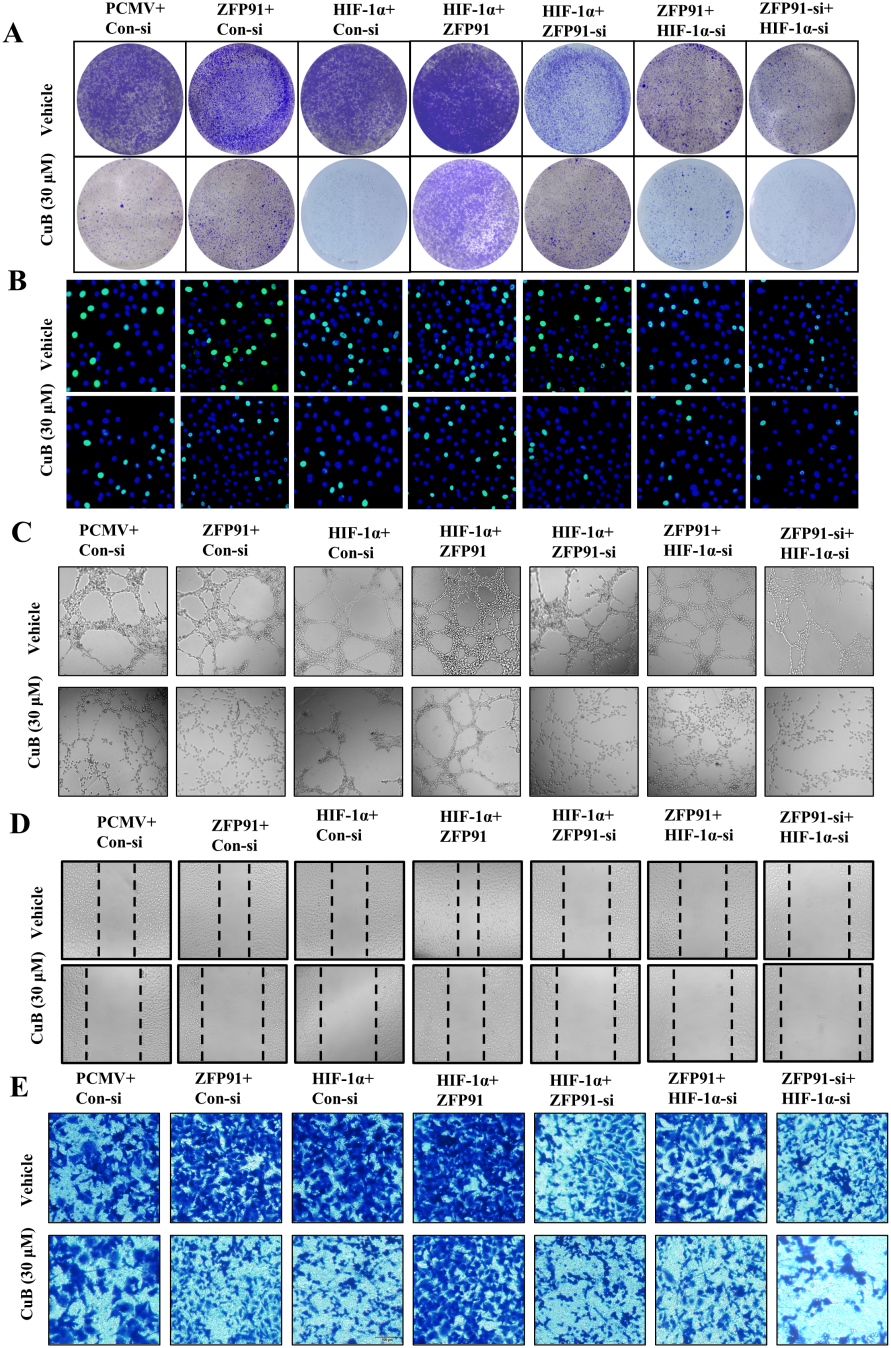
CuB inhibited the proliferation and invasion of A549 cells, as well as the angiogenesis and migration of HUVEC cells. **(A)** Colony formation assay to assess the number and size of A549 cell colonies. **(B)** EdU assay used to detect proliferating cells, with green fluorescence excitation at 488 nm. **(C)** Tubule formation assay to evaluate tubule formation in HUVEC cells on Matrigel (200× magnification). **(D)** Scratch assay to assess changes in cell migration, using light microscopy (200× magnification). **(E)** Representative image of invasion assay (200× magnification).

The overexpression of ZFP91 or HIF-1α increased the number of colonies, while the addition of CuB reversed this effect. Furthermore, the simultaneous silencing of ZFP91 and HIF-1α significantly reduced the number of cell colonies (**Figure 9A**). Edu assay similarly demonstrated that ZFP91 and HIF-1α play significant roles in cell proliferation. Additionally, CuB reduced the proliferation of A549 cells by inhibiting both ZFP91 and HIF-1α (**Figure 9B**). Angiogenesis, migration, and proliferation assays demonstrated that the overexpression of ZFP91 or HIF-1α significantly enhanced angiogenesis, cell migration, and invasion. In contrast, silencing ZFP91 or HIF-1α diminished these effects, while the simultaneous silencing of both ZFP91 and HIF-1α led to a more pronounced reduction in angiogenesis, cell migration, and invasion. On this basis, the migration, angiogenesis, and invasion of HUVEC were further inhibited by CuB (**Figures 9C–E**). Importantly, the overexpression of ZFP91, in the context of HIF-1α silencing, did not reverse the effects of the knockdown, suggesting that ZFP91 may function through HIF-1α. Conversely, the overexpression of HIF-1α, while ZFP91 was silenced, also failed to mitigate the effects of the knockdown, indicating that ZFP91 does not solely operate through HIF-1α.

### CuB inhibits growth of A549 cells in xenograft model

**Figure 10:**
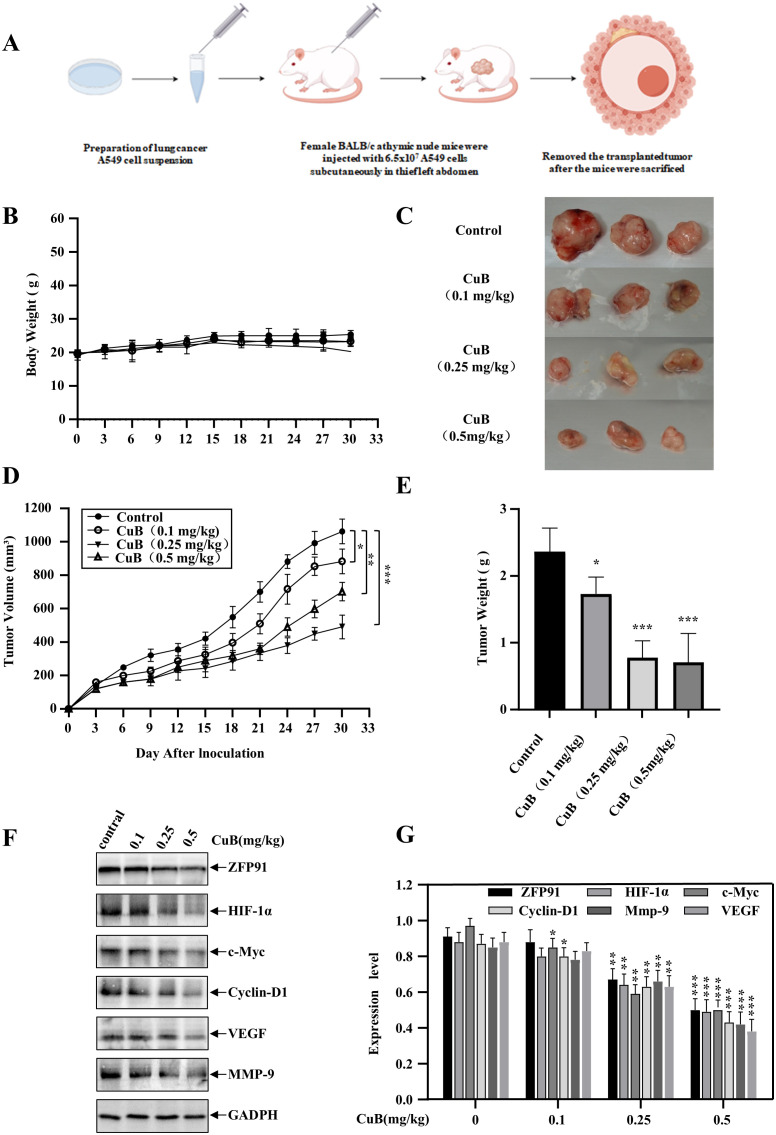
Therapeutic Effect of CuB on NSCLC *in vivo.* **(A)** Schematic diagram of xenograft model construction. **(B)** Change in mouse body weight. **(C-E)** Mice were euthanized, tumors excised, weighed, photographed, and their volumes measured. **(F, G)** Mouse colon tissues were homogenized and protein immunoblotting was performed to assess *in vivo* expression levels of ZFP91, HIF-1α, c-Myc, Cyclin D1, VEGF, and MMP-9. Data are mean ± SD, *p< 0.05, **p< 0.01, ***p<0.001 vs. Control group.

To further investigate the therapeutic effects of CuB on NSCLC, we established a xenograft model (**Figure 10A**). Our results indicated that CuB treatment significantly attenuated tumor burden (size/number) in mice without altering body weight (**Figures 10B-E**). Grinding tumor tissues and conducting Western blot experiments demonstrated that CuB effectively reduced the protein expression levels of ZFP91, HIF-1α, c-Myc, Cyclin D1, VEGF, and MMP-9 in tumor tissues (**Figures 10F, G**). These findings are consistent with our *in vitro* results.

## Discussion

Cancer is one of the most significant diseases threatening human life. Lung cancer ranks among the highest in terms of incidence, and the number of deaths attributed to lung cancer continues to rise each year (24). NSCLC is the predominant form of lung cancer, and recent advancements in treatment options have shown significant progress. However, individual variability, drug resistance, and high costs pose substantial challenges to their development (25). Therefore, it is essential to identify new therapeutic agents for the treatment of NSCLC.

CuB, a Cucurbitaceae-derived tetracyclic triterpenoid has been previously reported to have a significant therapeutic effect on lung cancer (26). In addition, cucurbitacin tablets containing CuB as the primary active ingredient have been utilized in China for the treatment of primary liver cancer. These results suggest that CuB is a viable option for the treatment of NSCLC. Our study confirmed the therapeutic effect of CuB on NSCLC. In comparison to previous studies, we discovered that CuB exerts its effects on NSCLC through the ZFP91/HIF-1α pathway. This finding enhances our understanding of the mechanism by which CuB treats NSCLC and provides a theoretical foundation for the development of CuB-based anticancer drugs. Compared to conventional anticancer drugs used in clinical practice, CuB offers several unique advantages. Extracted from cucurbitaceae plants, it is the most abundant member of the cucurbitacin family. Additionally, it is less expensive to extract and easier to synthesize (27). These results demonstrate the distinct advantages of CuB in the treatment of NSCLC and highlight its potential as an anti-lung cancer agent.

Zinc finger proteins are prevalent in eukaryotic genomes and perform diverse roles such as DNA recognition, RNA packaging, transcriptional activation, apoptosis regulation, protein folding/assembly, and lipid binding. These diverse functions are attributed to their unique structural characteristics (28). Previous studies have demonstrated that ZFP91 plays a significant role in various cancer types. Liu et al. indicated that ZFP91 functions as an atypical E3 ligase, promoting the degradation of the transcription factor E2F2, which may contribute to lung cancer treatment (29). Nevertheless, further studies are needed to elucidate the role of ZFP91 in lung cancer, particularly in NSCLC. Our results reveal an additional mechanism by which ZFP91 exerts its effects in NSCLC, through the PI3K/AKT/mTOR signaling pathway. This pathway influences the expression of HIF-1α, thereby contributing to the effects of ZFP91 in NSCLC. In addition to providing new mechanistic insights, our results indicate that CuB, a widely available natural compound, can regulate HIF-1α through ZFP91. This regulation inhibits the proliferation, migration, invasion, and angiogenesis of lung cancer cells, demonstrating promising therapeutic potential for NSCLC. Interestingly, previous studies conducted by our group have demonstrated that ZFP91 can activate HIF-1α expression through NF-κB (16). CuB can inhibit the expression of associated pathways in A549 cells *via* the NF-κB signaling pathway; therefore, we hypothesize that CuB may exert its effects by inhibiting the activation of HIF-1α through the suppression of ZFP91. However, this hypothesis requires validation through further experiments.

HIF-1α is a gene that plays a crucial role in cancer and is associated with various biological processe (10). Previous studies have demonstrated that CuB functions by inhibiting the protein synthesis of HIF-1α (30). Our research indicates that CuB modulates HIF-1α expression via the ZFP91 pathway. As an E3 ubiquitin ligase, ZFP91 is involved in protein degradation, indicating that the regulatory role of CuB on HIF-1α may also be linked to the ubiquitination process mediated by ZFP91. However, such speculation requires additional experimental evidence.

## Conclusion

**Figure 11:**
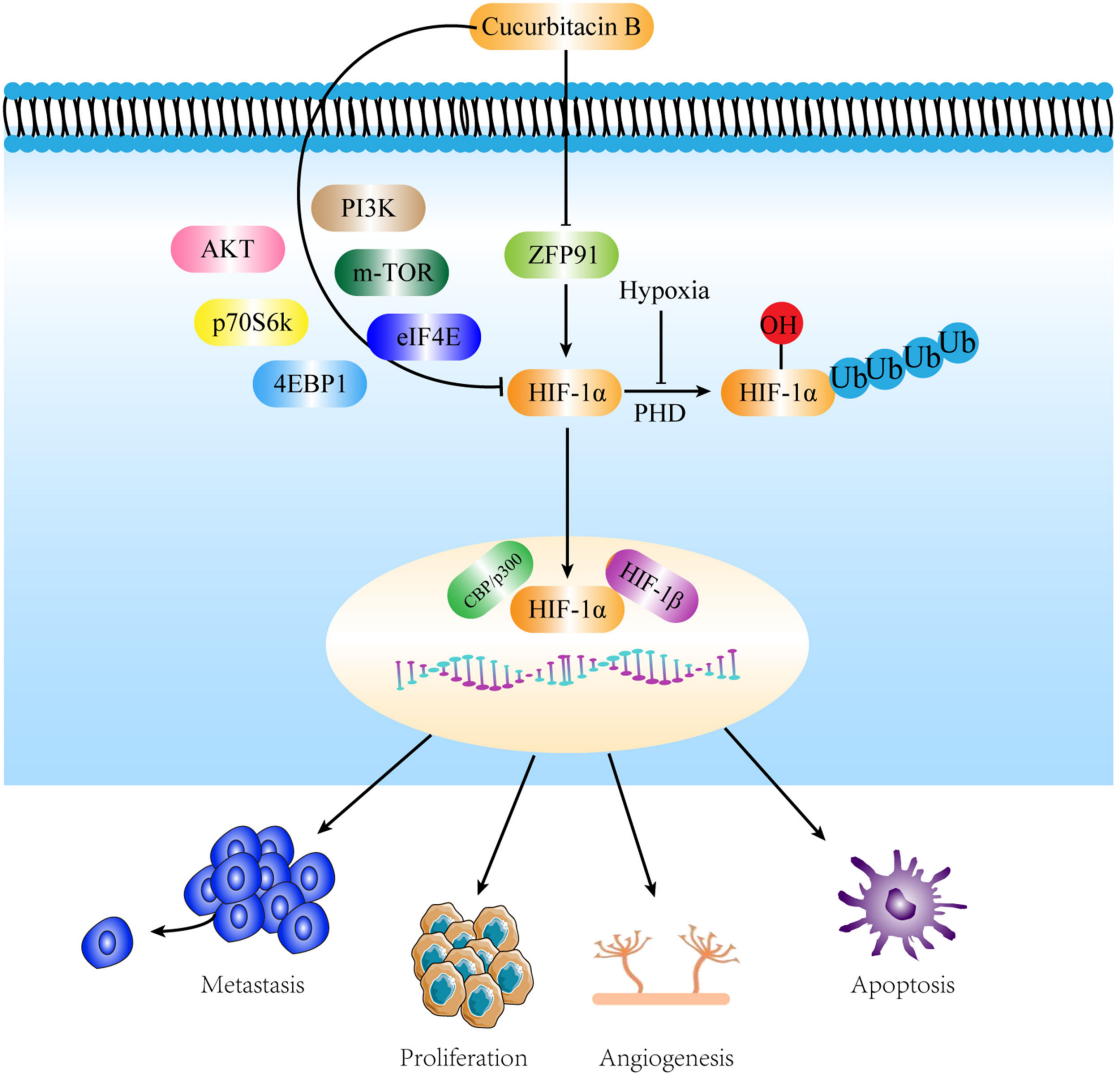
Mechanisms by which CuB attenuates NSCLC by inhibiting HIF-1α *via* ZFP91. CuB facilitated the nuclear entry of HIF-1α by inhibiting ZFP91, subsequently reducing the proliferation, migration, and angiogenesis of lung cancer cells. Additionally, CuB further suppressed HIF-1α expression by inhibiting the PI3K/AKT pathway, mTOR, p70S6K, 4EBP1, and eIF4E. These actions contribute to its anti- NSCLC effects.

Our experiments demonstrated for the first time that CuB inhibits the expression of HIF-1α and reduces the proliferation, migration, invasion, and angiogenesis of lung cancer cells by inhibiting ZFP91. In addition, CuB was able to inhibit the expression of PI3K/AKT, mTOR, p70S6K, eIF4E, and 4EBP1. Through these effects, CuB demonstrated a significant role in the treatment of NSCLC (**Figure 11**). Our preliminary results have demonstrated the anti-lung cancer effects of CuB and elucidated its mechanism of action, thereby providing a theoretical foundation for the development of CuB as a treatment for NSCLC.

## Data Availability

All relevant data is contained within the article: The original contributions presented in the study are included in the article/**Supplementary Material**, further inquiries can be directed to the corresponding author/s.
