# Correction: Cucurbitacin B inhibits HIF-1α and attenuates non-small cell lung cancer *via* ZFP91

**DOI:** 10.3389/fonc.2025.1684497

**Published:** 2025-09-04

**Authors:** Lei Song, Jing Han, Run Wang, Shen Cao, Yi Tai, Xinyu Wang, Yanjin Zheng, Shufeng Jin, Yue Xing, Hong Xiang Zuo, Ming Yue Li, Juan Ma, Xuejun Jin

**Affiliations:** ^1^ Key Laboratory of Natural Medicines of the Changbai Mountain, Ministry of Education, Molecular Medicine Research Center, College of Pharmacy, Yanbian University, Yanji, Jilin, China; ^2^ Affiliated Hospital of Yanbian University (Yanbian Hospital), Yanji, Jilin, China

**Keywords:** cucurbitacin B, non-small cell lung cancer, HIF-1α, ZFP91, anti-cancer

Affiliation “Key Laboratory of Natural Medicines of the Changbai Mountain, Ministry of Education, Molecular Medicine Research Center, College of Pharmacy, Yanbian University, Yanji, Jilin, China” was omitted for author Lei Song. This affiliation has now been added for author Lei Song.

Author Lei Song was erroneously assigned to affiliation “Department of Pharmacy, Yanbian University Hospital, Yanji, Jilin, China”. This affiliation has now been removed for author Lei Song.

There was a mistake in **Figure 3E** as published. The Western blot image for GAPDH was incorrectly uploaded. The corrected **Figure 3** appears below.

**Figure 3:**
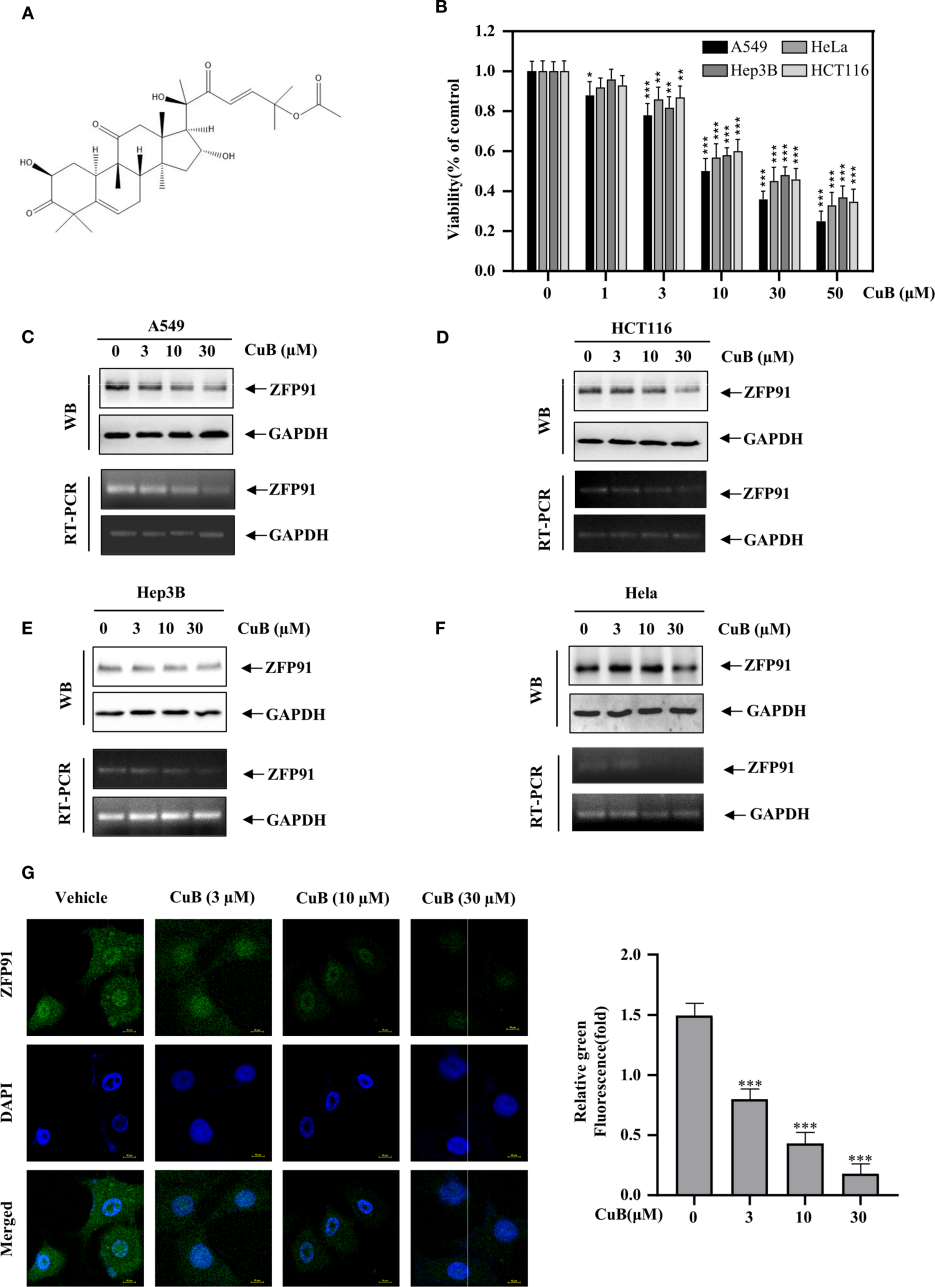
CuB inhibits the protein and mRNA expression of ZFP91. **(A)** Chemical Formula of CuB. **(B)** The MTT assay was used to evaluate CuB’s effect on A549 cell viability. **(C–F)** Effect of CuB on protein and mRNA expression of ZFP91 in A549, HCT116, Hep3B, and HeLa Cells. **(G)** Effect of CuB on ZFP91 fluorescence intensity in A549 cells. Original magnification: 600×. Data are represented as mean ± standard, * *p*<0.05, ** *p*<0.01, *** *p*<0.001 compared with Control group.

There was a mistake in the caption of **Figure 6B** as published. The label for IP was incorrect and should read “IP: HA-HIF-1α”. The corrected caption of **Figure 6** appears below.

**Figure 6:**
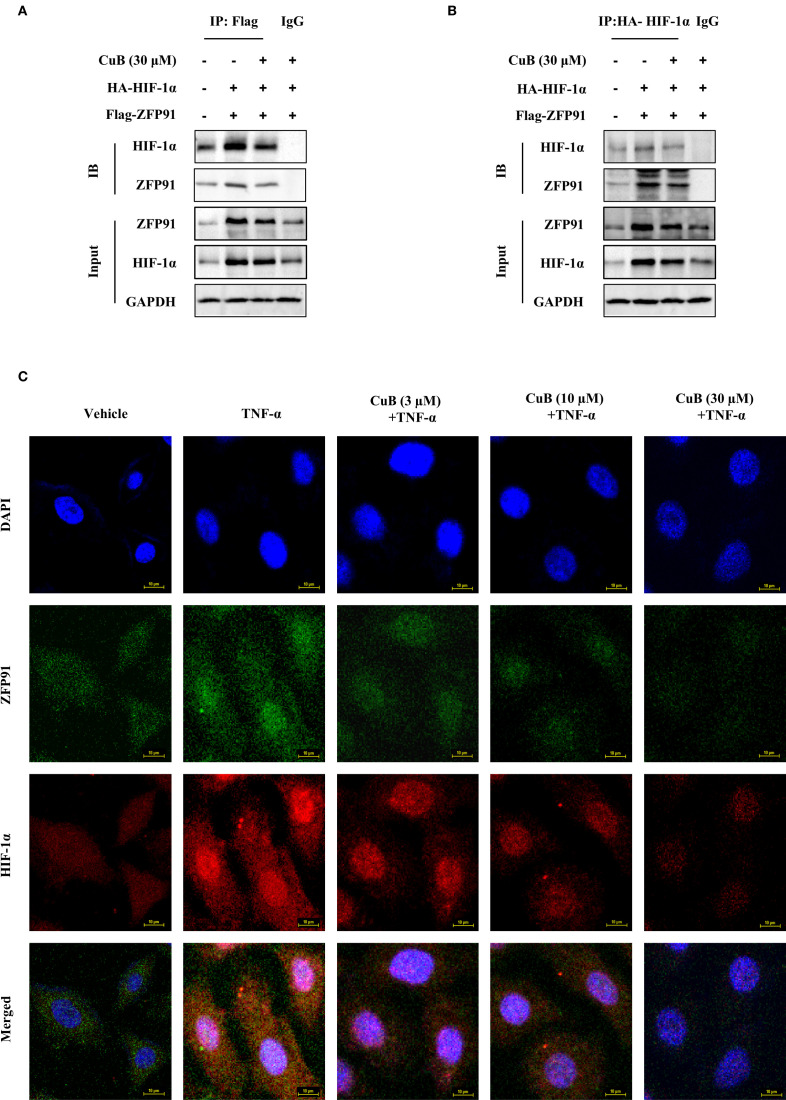
CuB inhibited the interaction between ZFP91 and HIF-1α. **(A, B)** A549 cells were transfected with IP: HA-HIF-1α and Flag-ZNF91 plasmids for. Co-immunoprecipitation was then performed to pull down either Flag-ZNF91 or IP: HA-HIF-1α. **(C)** Co-localization of ZFP91 with HIF-1α within the cells was detected using immunofluorescence. Magnification: 600×.

Due to a clerical error by the author, “HIF-1α” was mistakenly written as “HID-1a” and should be corrected to “HIF-1α.”.

A correction has been made to the section **Results**, *CuB inhibits the expression of HIF-1α through ZFP91*:

“In addition, co-immunoprecipitation experiments demonstrated an interaction between ZFP91 and HIF-1α; however, the addition of CuB was found to attenuate this interaction (Figures 6A, B).”

The original version of this article has been updated.

