# Supplementary material for: Cucurbitacin B inhibits HIF-1α and attenuates non-small cell lung cancer via ZFP91
Source: Front Oncol. 2025 Aug 11;15:1607264. doi: 10.3389/fonc.2025.1607264 (PMC12375561; doi:10.3389/fonc.2025.1607264)
Supplement: Supplementary file 1 [file DataSheet1.docx]

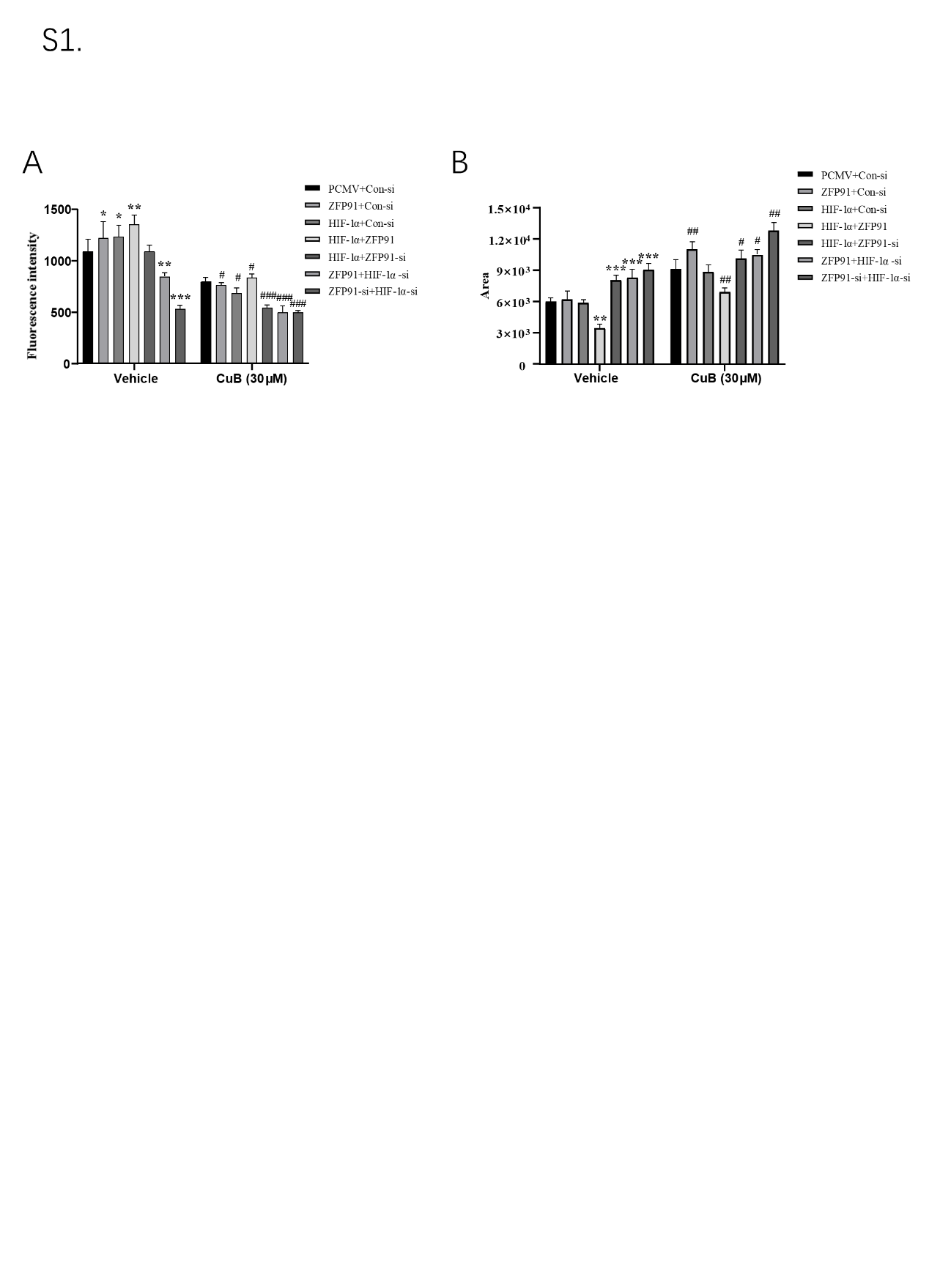


**Supplement Figure Legends**

**Fig. S1** (A) Statistical analysis of Fig. 9B. (B) Statistical analysis of Fig. 1D. Three independent experiments were performed and the mean ± standard deviation of three independent experiments, **p* < 0.05, ***p* < 0.01，****p* < 0.001 compared to PCMV-Con-si group, *#p* < 0.05, *##p* < 0.01，*###p* < 0.001 compared to PCMV-Con-si + CuB group.
